# Osteoporosis and prostate cancer

**DOI:** 10.1038/sj.bjc.6601191

**Published:** 2003-08-26

**Authors:** T Dhillon, J Waxman

**Affiliations:** 1 Department of Cancer Medicine, Faculty of Medicine, Imperial College of Science, Technology & Medicine, Hammersmith Campus, Du Cane Road, London W12 0NN, UK

Prostate cancer has just become the most commonly registered cancer of males in the United Kingdom, overtaking lung cancer in the registration sweepstakes, and is the second most common cause of male cancer deaths. Deaths have trebled over the last 30 years, plateauing in the mid-1990s but recently increasing again (Office for National Statistics, 2001).

Locally advanced and metastatic prostate cancer is treated with antiandrogens and the importance of the side effects of treatment have become more significant as the incidence of this malignancy has increased. These side effects from treatment range from hot flushes, impotence, breast tenderness and enlargement, and diarrhoea to the physically more significant side effects, which include anaemia, loss of muscle bulk, neurological dysfunction, and osteoporosis. All of these side effects occur as a consequence of androgen deficiency. We would argue that the development of osteoporosis is the most important of these side effects and that we need to be more proactive in our management of this condition.

The risk of osteoporotic fracture as a consequence of antiandrogen therapy has been described in five studies. In a group of 235 patients with prostate cancer, the cumulative risk of fracture at 7 years was 28% in 59 castrated men and 1% in 176 men treated with a GnRH agonist (Daniell, 1997). In a second study, 9% of 224 men treated with an LHRH agonist had a fracture over a median follow-up of 22 months; seven of these men had fractures resulting from osteoporosis, eight men from trauma, and five of mixed aetiology (Townsend *et al*, 1997). In a third study of 218 men, 6% had an osteoporotic fracture during 6 or more months treatment with anti-androgen therapy (Hatano *et al*, 2000); in a fourth study, 20% of 181 men treated for 10 years had osteoporotic fractures (Oefelein *et al*, 2001). Fracture rates were described in 429 men with prostate cancer treated by bilateral orchiectomy. There were 267 fractures in 161 men. The increased risk due to osteoporosis was reported as a standard incidence ratio of 3.50 (95% CI 2.71–4.43) (Melton *et al*, 2003).

The increased risk of fracture in these men is due to accelerated loss of bone mineral density and is of early onset. Decreases of 6.6% at the lumbar spine have been seen only after 6 months of treatment and decreases of 6% at the femoral neck after 18 months treatment (Diamond *et al*, 1998). In a French study, changes in bone mineral density were evaluated in 12 men with prostate cancer treated with triptorelin. Men with metastases, radiographic evidence of osteoporosis, or pre-existing conditions associated with accelerated bone loss were excluded from the study. Bone mineral density was measured at baseline, 6, 12, and 18 months. Bone mineral density of the lumbar spine and femoral neck tended to decrease from month 6, but only reached statistical significance at 18 months (Maillefert *et al*, 1999). In an American study, changes in bone mineral density were evaluated in 26 men with prostate cancer treated by orchiectomy or with GnRH agonists. Bone mineral density was measured at 6–42 months. The average bone mineral density of the hip decreased by 7.6% during the first orchiectomy, and similar rates of bone loss were observed among men treated with the GnRH agonists. Rates of bone loss were highest among inactive and obese men (Daniell *et al*, 2000).

These significant changes in bone mineralisation can be prevented. This has been shown by a recent study, where patients with locally advanced or recurrent nonmetastatic prostate cancer receiving leuprolide were randomised to receive pamidronate given every 3 months for 1 year. In the bisphosphonate group bone mineral density did not decrease, while in the control group mineralisation decreased by 3.3% in the lumbar spine and 2.1% at the hip (Smith *et al*, 2001). This benefit has been confirmed in an Australian study (Diamond *et al*, 2001) and in an American study where 106 patients were randomised to receive 3 monthly Zoledronic acid for 1 year (Smith *et al*, 2003a). An alternative approach might be to use an antiandrogen without adverse effects on bone. Bicalutamide monotherapy increases serum levels of testosterone and oestradiol, indicating that osteoporosis might be less likely than with other antiandrogens. Urinary markers of bone turnover in bicalutamide-treated patients were higher than in gonadotrophin-releasing hormones against treated patients (Smith *et al*, 2003b). However, there have been no studies that have assessed bone mineralisation densities in patients treated with bicalutamide as compared with other antiandrogens. In clinical practice, bicalutamide is frequently given with a gonadotrophin-releasing hormone agonist and the effects of this combination on bone turnover are not known.

So, it would appear that there is a clear way to limit the effects of antiandrogen treatment on bone and that is by giving bisphosphonate treatment prophylactically. What is unclear is the bisphosphonate dosage regimen to be used. There are new, highly effective third-generation bisphosphonates curr-ently available and it may be that a limited trial is required to investigate this issue of dosage and frequency of bisphosphonate administration.

## References

[bib1] Daniell HW (1997) Osteoporosis after orchiectomy for prostate cancer. J Urol 157: 439–444 8996327

[bib2] Daniell HW, Dunn SR, Ferguson DW, Lomas G, Niazi Z, Stratte PT (2000) Progressive osteoporosis during androgen deprivation therapy for prostate cancer. J Urol 163: 181–186 10604342

[bib3] Diamond T, Campbell J, Bryant C, Lynch W (1998) The effect of combined androgen blockade on bone turnover and bone mineral densities in men treated for prostate carcinoma: longitudinal evaluation and response to intermittent cycle etidronate therapy. Cancer 83: 561–566 9781950

[bib4] Diamond TH, Winters J, Smith A, De Souza P, Kersley JH, Lynch WJ, Bryant C (2001) The antiosteoporosis efficacy of intravenous pamidronate in men with prostate carcinoma receiving combined androgen blockade: a double blind, randomized, placebo-controlled crossover study. Cancer 92: 1444–1450 11745221 10.1002/1097-0142(20010915)92:6<1444::aid-cncr1468>3.0.co;2-m

[bib5] Hatano T, Oishi Y, Furuta A, Iwamuro S, Tashiro K (2000) Incidence of bone fractures in patients receiving LH-releasing hormone agonists for prostate cancer. Br J Uro Int 86: 449–452 10.1046/j.1464-410x.2000.00774.x 10971270

[bib6] Maillefert JF, Sibilia J, Michel F, Saussine C, Javier RM, Tavernier C (1999) Bone mineral density in men treated with synthetic gonadotropin-releasing hormone agonists for prostate carcinoma. J Urol 161: 1219–1222 10081873

[bib7] Melton III LJ, Alothman KI, Khosla S, Achenbach SJ, Oberg AL, Zincke H (2003) Fracture risk following bilateral orchiectomy. J Urol 169 (5): 1747–1750 12686824 10.1097/01.ju.0000059281.67667.97

[bib8] Oefelein MG, Ricchuiti V, Conrad W, Seftel A, Bodner D, Goldman H, Resnick M (2001) Skeletal fracture associated with androgen suppression induced osteoporosis: the clinical incidence and risk factors for patients with prostate cancer. J Urol 166: 1724–1728 11586210 10.1016/s0022-5347(05)65661-3

[bib9] Office for National Statistics (2001), Website address: www.statisticsgov.uk/statbase/explorer.asp

[bib10] Smith MR, Eastham J, Gleason DM, Shasha D, Tchekmedyian S, Zinner N (2003a) Randomized controlled trial of zoledronic acid to prevent bone loss in men receiving androgen deprivation therapy for nonmetastatic prostate cancer. J Urol 169 (6): 2008–2012 12771706 10.1097/01.ju.0000063820.94994.95

[bib11] Smith MR, Fallon MA, Goode MJ (2003b) Cross-sectional study of bone turnover during bicalutamide monotherapy for prostate cancer. Urology 61 (1): 127–131 12559282 10.1016/s0090-4295(02)02006-x

[bib12] Smith MR, McGovern FJ, Zietman AL, Fallon MA, Hayden DL, Schoenfeld DA, Kantoff PW, Finkelstein JS (2001) Pamidronate to prevent bone loss during androgen-deprivation therapy for prostate cancer. N Eng J Med 345 (13): 948–954 10.1056/NEJMoa010845 11575286

[bib13] Townsend MF, Sanders WH, Northway RO, Graham Jr SD (1997) Bone fractures associated with LH-releasing hormone agonists used in the treatment of prostate carcinoma. Cancer 79: 545–550 9028366 10.1002/(sici)1097-0142(19970201)79:3<545::aid-cncr17>3.0.co;2-3

